# Potential Usefulness of Urinary Hepcidin Measurement for Iron Deficiency Anemia in Female Athletes

**DOI:** 10.1002/ejsc.70198

**Published:** 2026-05-22

**Authors:** Haruo Hanawa, Hiromi Inaba, Fumi Hoshino, Mutsuaki Edama, Chie Sekine, Takanori Kikumoto, Tomonobu Ishigaki, Yuiko Matsuura, Go Omori

**Affiliations:** ^1^ Department of Clinical Engineering and Medical Technology Niigata University of Health and Welfare Niigata Japan; ^2^ Athlete Support Research Center Niigata University of Health and Welfare Niigata Japan; ^3^ Department of Health and Nutrition Niigata University of Health and Welfare Niigata Japan; ^4^ Department of Physical Therapy Niigata University of Health and Welfare Niigata Japan; ^5^ Department of Physical Therapy Faculty of Medical Science Teikyo University of Science Adachi Japan; ^6^ Department of Physical Education College of Humanities and Sciences Nihon University Chiyoda Japan; ^7^ Department of Health and Sports Niigata University of Health and Welfare Niigata Japan

**Keywords:** anemia, athlete, hepcidin, iron deficiency, noninvasive screening

## Abstract

Hepcidin is a key regulator of iron metabolism and has been proposed as a biomarker for detecting iron deficiency. This study examined serum hepcidin and non‐invasively measured urinary hepcidin in 260 female athletes to assess their potential utility in evaluating iron status. Urinary hepcidin/creatinine showed a strong correlation with serum hepcidin and was positively associated with ferritin, suggesting that it reflects underlying iron dynamics. Iron‐related measures, including hepcidin, showed expected differences across iron‐status categories, consistent with physiological changes from iron repletion to iron deficiency anemia. In diagnostic analyses, both serum hepcidin and urinary hepcidin/creatinine demonstrated good accuracy for identifying iron deficiency including iron deficiency anemia. Serum hepcidin yielded AUCs of approximately 0.85–0.90, while urinary hepcidin/creatinine showed AUCs around 0.85–0.88. Internal validation produced consistent confidence intervals, suggesting stable estimates. At the Youden‐optimal cutoff, both markers showed comparable performance, with sensitivities of 0.74–0.91, specificities of 0.73–0.92, and high negative predictive values (0.83–0.97). These cutoff values were derived from internally validated analyses and should be considered preliminary, requiring external validation before broader clinical implementation. These findings suggest that hepcidin‐based measures may assist in ruling out iron deficiency or guiding triage decisions. Because urinary hepcidin can be obtained non‐invasively and demonstrated performance similar to serum hepcidin, it may represent a practical screening option in athletic populations, although further external validation is needed.

## Highlights

- Urinary hepcidin correlated strongly with serum hepcidin and ferritin, reflecting systemic iron status.
- Serum and urinary hepcidin levels decreased progressively from iron repletion to iron deficiency anemia.
- Both serum hepcidin and urinary hepcidin/creatinine ratio showed good diagnostic performance for iron deficiency and IDA.
- Internal validation using athlete‐level cluster bootstrap demonstrated robust diagnostic performance.
- The urinary hepcidin/creatinine ratio may serve as a practical noninvasive biomarker for screening iron deficiency in athletes.

## 1 Introduction

Women are more susceptible to iron deficiency and anemia than men due to menstruation, and female athletes are also more susceptible than women of the same age (Dubnov et al. 2006). Increased iron demand due to increased muscle mass, iron loss through sweat, bleeding from the digestive tract and urinary tract, and decreased oral intake due to training are thought to be factors that make athletes more susceptible to iron deficiency and anemia than the general population (Damian et al. 2021). Iron deficiency and anemia are important issues for female athletes because they affect their performance (Solberg and Reikvam 2023). Therefore, early detection and appropriate treatment are necessary, and medical checkups are recommended (Clénin et al. 2015). Increasing the frequency of medical checkups would be beneficial for early detection, but consideration must also be given to the burden on athletes. Furthermore, junior high schools, high school, and university schools often do not have full‐time staff capable of taking blood samples, making it difficult to conveniently draw blood samples on‐campus to test for iron deficiency and anemia for the many female students who belong to sports clubs. For these reasons, simpler, non‐invasive methods for detecting iron deficiency and anemia, such as transcutaneous Hb measurement, are desirable (Bhat et al. 2016; Paksu et al. 2016).

Hepcidin, discovered as an antimicrobial peptide in urine, is a peptide hormone that plays a central role in regulating iron metabolism (Hunter et al. 2002; Park et al. 2001). Hepcidin, which is mostly produced in the liver, has a small molecular weight of approximately 2.8 kDa and is easily filtered through the glomerulus, making it easily detectable in urine (Wolff et al. 2013). Hepcidin inhibits ferroportin, thereby suppressing iron absorption from the gastrointestinal tract and iron release from macrophages and other cells (Nemeth, Tuttle, et al. 2004). Increased blood transferrin iron promotes hepcidin production, but conversely, low transferrin iron reduces hepcidin production (Bartnikas et al. 2011; Gao et al. 2009). Furthermore, anemia increases erythropoietin, which in turn increases bone marrow erythroferrone and other hormones, further reducing hepcidin levels (Ganz 2019b; Kautz et al. 2014). In other words, absolute iron deficiency and anemia reduce hepcidin production. On the other hand, inflammation is known to increase inflammatory cytokines such as IL‐6, which enhance hepcidin expression (Nemeth, Rivera, et al. 2004). It is believed that inflammatory anemia results from impaired iron utilization due to excessive production of hepcidin (Ganz 2019a).

In this study, we analyzed data on iron deficiency and anemia from health checkups of female athletes in Niigata Prefecture. We measured urinary hepcidin using urine, which can be collected non‐invasively even in areas where there are no staff available to draw blood, and examined its significance as a biomarker for iron deficiency and anemia.

## 2 Materials and Methods

### 2.1 Subjects

**FIGURE 1:**
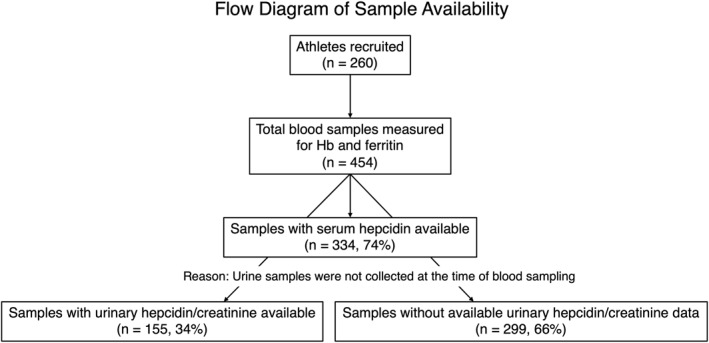
Flow diagram of study population and sample availability. Urinary hepcidin measurements were available for 155 of 454 samples (34%), as urine samples were not collected at the time of blood sampling.

A total of 260 female athletes (454 samples) belonging to training clubs in Niigata Prefecture who underwent medical checkups from 2021 to 2024 were analyzed. As shown in Figure 1, serum samples were available for 334 samples, and urinary hepcidin/creatinine measurements were available for 155 samples. The training clubs to which the athletes belong are shown in Table 1. Because several sports had small sample sizes, sports were grouped into four broader categories based on training characteristics: team sports, endurance sports, power/sprint sports, and skill/aesthetic sports. The age of the athletes was 19.0 ± 2.6 years (mean ± standard deviation). After both written and verbal instruction and information on the study, participants signed informed consent to participate. Ethics approval for this study was obtained through the Human Research Ethics Board of Niigata University of Health and Welfare (protocol number 18637–210618) in accordance with the Declaration of Helsinki.

**TABLE 1:** Sport club.

Club	No.	%
Basketball	47	18.1
Soccer	114	43.8
Volleyball	29	11.2
Track and field (long distance)	22	8.5
Track and field (sprint)	7	2.7
Track and field (throwing)	2	0.8
Swimming	13	5.0
table tennis	5	1.9
Tennis	4	1.5
Dance	2	0.8
Cheerleader	10	3.8
Snowboarding	3	1.2
Naginata	2	0.8
Total	260	100

### 2.2 Test Items

Red blood cell count (RBC), hemoglobin (Hb), hematocrit (Ht), serum iron (Fe), unsaturated iron‐binding capacity (UIBC), serum ferritin (Ft), serum hepcidin, urinary hepcidin and urinary creatinine (Cr) were measured. Transcutaneous Hb was measured 231 times using a Pronto (Masimo, Tokyo, Japan) and 171 times using an Astrim Fit (Sysmex, Kobe, Japan). Blood samples were measured for RBC, Hb, Ht, Fe, UIBC, and Ft within 3 h after collection, and serum for serum hepcidin measurement was stored at −80°C. Urine samples were centrifuged within 6 h after collection to remove sediment, and the supernatant was stored at −80°C. Urinary hepcidin measurements were available for 155 of 454 samples (34%), as urine samples were not collected at the time of blood sampling. Hepcidin was measured in serum (*n* = 334) and urine (*n* = 155) samples that were preserved, using a Human Hepcidin Immunoassay ELISA kit (R&D Systems, Minneapolis, MN). The limits of detection (LOD) and quantification (LOQ) for each assay were defined according to the manufacturer's specifications. For serum Ft, one value below the LOD (1 ng/mL) was observed among 454 samples and was imputed as half the LOD (0.5 ng/mL) for statistical analyses. For serum hepcidin, eight values below the LOD (0.0325 ng/mL) were identified among 334 samples and were replaced with half the LOD (0.0163 ng/mL). One value exceeded the upper limit of quantification (LOQ) and was assigned the LOQ value (109.151 ng/mL). For urinary hepcidin normalized to creatinine (hepcidin/Cr), the LOD and LOQ were 0.0874 ng/mL and 505.8 ng/mL, respectively. No values were below the LOD or above the LOQ among the 155 samples analyzed; therefore, no substitution was required. Values below the LOD were replaced with half the LOD, a commonly used approach for handling left‐censored biomarker data. Assay performance of the hepcidin ELISA was evaluated by determining intra‐ and inter‐assay coefficients of variation (CVs), dilution linearity, spike‐and‐recovery, and freeze–thaw stability. Intra‐ and inter‐assay CVs were determined using repeated measurements of pooled urine samples. Dilution linearity was evaluated using serial two‐fold dilutions (10×–80×), which demonstrated acceptable linearity across the tested range. Spike‐and‐recovery and freeze–thaw stability testing demonstrated acceptable assay performance. Detailed validation results are summarized in Supporting Information S1: Table S1.

### 2.3 Proportion of Anemia and Iron Deficiency Stages

Using the stage classification of the Swiss Society of Sports Medicine, anemia and iron deficiency stages were classified as normal, non‐anemic iron deficiency (NAID), iron deficiency with microcytosis and hypochromia (IDMH), iron deficiency anemia (IDA), and non‐iron deficiency anemia (NIDA) (Clénin et al. 2015). According to this classification, an Hb level of less than 12.0 g/dL is defined as anemia, and an Ft level of less than 30 ng/mL is defined as iron deficiency. In cases where blood samples were taken multiple times for the same case, the more severe results were used to analyze the proportion of anemia and iron deficiency stages for 260 athletes, and the proportion of stages for all 454 samples was also analyzed.

### 2.4 Oral Iron Treatment

Twenty‐three athletes who were judged by a doctor to be suitable for oral iron treatment based on IDA or IDMH and who provided their consent were prescribed pharmaceutical iron supplements at a medical institution. After taking oral iron supplements of 25–100 mg per day for more than one month, blood samples were taken and Hb levels were compared before and after treatment.

### 2.5 Statistical Processing

Statistical analyses were performed using SPSS version 29.0 for Mac (SPSS Inc., Chicago, IL) and R software (version 2026.01.1 + 403; R Foundation for Statistical Computing, Vienna, Austria). Continuous variables are presented as medians with interquartile ranges or mean ± standard deviation, as appropriate. Categorical variables are presented as counts and percentages. Continuous variables were compared using the Wilcoxon rank‐sum test. Categorical variables were compared using Pearson's chi‐squared test. The mean Hb concentrations before and after treatment were compared using the paired Student's *t* test. To assess potential selection bias due to missing urinary hepcidin measurements, key baseline characteristics, including age, Hb, Ft, C‐reactive protein (CRP), serum creatinine, estimated glomerular filtration rate (eGFR), and sport category, were compared between samples with and without urinary hepcidin data. Furthermore, factors associated with urinary hepcidin availability were evaluated using Firth's penalized logistic regression analysis including age, Hb, Ft, and sport category. Sensitivity analyses were subsequently conducted using inverse probability weighting (IPW). Because multiple samples were obtained from the same athletes, all analyses accounted for within‐athlete clustering. Associations between biomarkers were evaluated using mixed‐effects linear regression models with random intercepts for each athlete. The following models were specified: log urinary hepcidin/creatinine as a function of log serum hepcidin; log Ft as a function of log serum hepcidin; log Ft as a function of log urinary hepcidin/creatinine; and venous Hb as a transcutaneous Hb (Pronto and Astrim). Results are reported as regression coefficients (*β*) with standard errors (SE), 95% confidence intervals (CI), and *p*‐values.

Iron status stages were categorized into four groups based on Ft, transferrin saturation (TSAT), and Hb levels: Iron replete: Ft ≥ 30 ng/mL; Low Ft: Ft < 30 ng/mL, TSAT ≥ 20%, Hb ≥ 12 g/dL; Low Ft + low TSAT: Ft < 30 ng/mL, TSAT < 20%, Hb ≥ 12 g/dL; Iron deficiency anemia (IDA): Ft < 30 ng/mL and Hb < 12 g/dL.

Stage‐stratified distributions of MCV, MCH, log serum hepcidin, and log urinary hepcidin/creatinine were compared using the Kruskal–Wallis test, followed by pair wise Wilcoxon rank‐sum tests with Bonferroni correction. Diagnostic performance was assessed using receiver operating characteristic (ROC) curve analysis. To account for repeated measurements and to avoid optimistic bias, internal validation was performed using athlete‐level cluster bootstrap resampling (1000 iterations). In each bootstrap iteration, athletes were sampled with replacement, and optimal cutoff values were determined using the Youden index in the bootstrap sample and evaluated in out‐of‐bag athletes. Area under the curve (AUC), sensitivity, specificity, positive predictive value (PPV), negative predictive value (NPV), and likelihood ratios (LR+ and LR−) were calculated with 95% confidence intervals derived from bootstrap distributions. For comparison, conventional analyses without internal validation were performed and are presented in the Supporting Information S1. To evaluate potential confounding by inflammation and renal function, sensitivity analyses were performed excluding samples with C‐reactive protein (CRP) > 0.3 mg/dL or estimated glomerular filtration rate (eGFR) < 60 mL/min/1.73 m^2^. All statistical tests were two‐sided, and *p* values < 0.05 were considered statistically significant.

## 3 Results

### 3.1 Anemia and Iron Deficiency Stage

**FIGURE 2:**
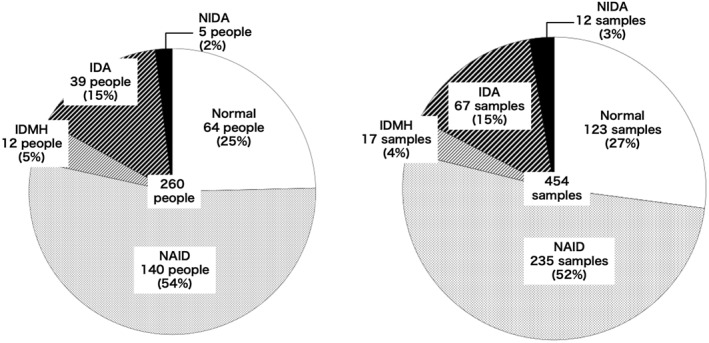
Anemia and iron deficiency stage in 260 athletes and 454 samples.

Table 2 shows the blood findings of 260 athletes and 454 samples. The data for each athlete represent the most severe anemia and iron deficiency in that individual. The proportions of anemia and iron deficiency were normal in 64 athletes (25%), NAID in 140 athletes (54%), IDMH in 12 athletes (5%), IDA in 39 athletes (15%), and NIDA in 5 athletes (2%). The proportions of anemia and iron deficiency among the 454 samples were normal in 123 samples (27%), NAID in 235 samples (52%), IDMH in 17 samples (4%), IDA in 67 samples (15%), and NIDA in 12 samples (3%) (Figure 2).

**TABLE 2:** Blood findings (260 peoples^a^, 454 samples).

	260 peoples	454 samples
Hemoglobin, g/dL	12.8 ± 1.1	12.8 ± 1.0
MCV, fl	90.3 ± 4.3	90.5 ± 4.1
MCH, pg	29.4 ± 1.7	29.6 ± 1.6
MCHC, %	32.6 ± 1.0	32.7 ± 1.0
Serum iron, μg/dL	83.8 ± 37.6	87.6 ± 39.5
Ferritin, ng/mL	23.3 ± 11.6	25.6 ± 20.1

*Note:* Continuous variables are shown as means ± SD.

^a^ When blood samples were taken multiple times for the same case, the more severe result was used.

### 3.2 Baseline Characteristics by Urine Sample Availability

Urinary hepcidin measurements were available in a subset of samples. Comparisons between samples with and without urinary hepcidin data showed no significant differences in age, Hb, Ft, CRP, serum creatinine, or eGFR, whereas the distribution of sport categories differed significantly, suggesting a potential risk of selection bias (Table 3). Firth's penalized logistic regression analysis showed that age and sport category were significantly associated with the availability of urinary hepcidin measurements, indicating potential selection bias related to athlete characteristics (Supporting Information S1: Table S2). Supporting Information S1: Figure S1 shows the distribution of stabilized inverse probability weights used in the sensitivity analyses, illustrated as a histogram derived from the propensity score model for urinary hepcidin availability.

**TABLE 3:** Baseline characteristics.

Variable	Urine.available^a^	Urine.not.available^a^	*p*‐value^b^
Age, years	19 [18–20] (*n* = 155)	19 [18–20] (*n* = 299)	0.637
Hemoglobin, g/dL	13 [12.4–13.4] (*n* = 155)	12.8 [12.15–13.5] (*n* = 299)	0.305
Ferritin, ng/mL	20 [11–30] (*n* = 155)	22 [12–34] (*n* = 299)	0.192
CRP, mg/dL	0.01 [0–0.03] (*n* = 120)	0.01 [0–0.03] (*n* = 21)	0.572
Serum creatinine, mg/dL	0.71 [0.66–0.77] (*n* = 120)	0.68 [0.64–0.76] (*n* = 21)	0.140
eGFR, mL/min/1.73m2	88.6 [80.7–97.5] (*n* = 120)	92.5 [84.4–100.2] (*n* = 21)	0.399
Sport group distribution			< 0.001
Team	104 (67.1%)	250 (83.6%)	
Endurance	22 (14.2%)	41 (13.7%)	
Power	9 (5.8%)	0 (0%)	
Skill	20 (12.9%)	8 (2.7%)	

^a^ Median (Q1, Q3); *n* (%).

^b^ Wilcoxon rank sum test; Pearson's Chi‐squared test.

### 3.3 Hepcidin as an Indicator of IDA and Iron Deficiency

**FIGURE 3:**
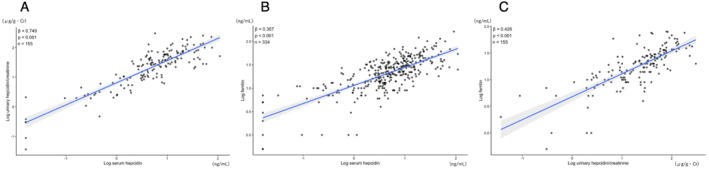
Associations between biomarkers. (A) Relationship between log serum hepcidin and log urinary hepcidin/creatinine. (B) Relationship between log serum hepcidin and log Ft. (C) Relationship between log urinary hepcidin/creatinine and log Ft. Lines represent fitted values from mixed‐effects linear regression models with random intercepts for each athlete. Shaded areas indicate 95% confidence intervals.

**FIGURE 4:**
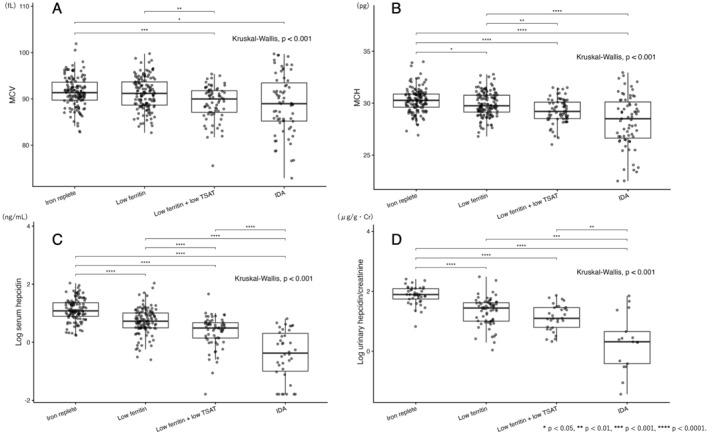
Stage‐stratified distributions of MCV (A), MCH (B), log serum hepcidin (C), and log urinary hepcidin/creatinine (D). Athletes were classified into iron replete, low Ft, low Ft plus low TSAT, and iron deficiency anemia groups. Overall differences were assessed using the Kruskal–Wallis test. Pairwise comparisons were performed using the Wilcoxon rank‐sum test with Bonferroni correction.

Mixed‐effects linear regression analyses accounting for within‐athlete clustering demonstrated significant positive associations between biomarkers. Log urinary hepcidin/creatinine was strongly associated with log serum hepcidin (*β* approximately 0.75, *p* < 0.001). Log Ft was also significantly associated with log serum hepcidin (*β* approximately 0.36, *p* < 0.001) and with log urinary hepcidin/creatinine (*β* approximately 0.43, *p* < 0.001). These associations are summarized in Table 4 and illustrated in Figure 3. Distributions of MCV, MCH, log serum hepcidin, and log urinary hepcidin/creatinine across iron status stages are shown in Figure 4. Significant differences across the four groups were observed for all variables (Kruskal–Wallis test, all *p* < 0.001). Pairwise comparisons showed progressive reductions in MCH, serum hepcidin, and urinary hepcidin from iron replete to iron deficiency anemia.

**TABLE 4:** Associations between biomarkers evaluated using mixed‐effects linear regression models.

Predictor	*β*	SE	95% CI	*p*‐value
Log serum hepcidin → log urine hepcidin	0.749	0.035	0.680–0.818	< 0.001
Log serum hepcidin → log ferritin	0.357	0.020	0.319–0.396	< 0.001
Log urine hepcidin → log ferritin	0.426	0.030	0.368–0.485	< 0.001

**FIGURE 5:**
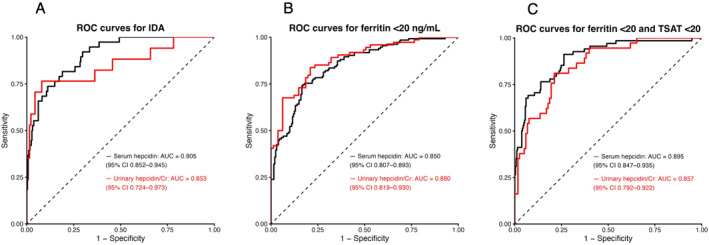
Receiver operating characteristic (ROC) curves for detection of iron deficiency and iron deficiency anemia. (A) Iron deficiency anemia (IDA). (B) Ft < 20 ng/mL. (C) Ft < 20 ng/mL with transferrin saturation (TSAT) < 20%. ROC curves for serum hepcidin (black) and urinary hepcidin corrected by creatinine (red) are shown. ROC curves represent apparent diagnostic performance. Area under the curve (AUC) values and 95% confidence intervals were estimated using athlete‐level cluster bootstrap resampling.

In diagnostic performance analyses, serum hepcidin demonstrated excellent discrimination for iron deficiency anemia (IDA), whereas urinary hepcidin/creatinine showed good performance, particularly for detecting Ft < 20 ng/mL (Figure 5) (Supporting Information S1: Table S3). Internal validation using athlete‐level cluster bootstrap yielded AUC values that were consistent with the apparent performance, although confidence intervals were wider, reflecting more conservative and robust estimates (Table 5). For example, the internally validated AUC for serum hepcidin in detecting IDA was approximately 0.90, while urinary hepcidin/creatinine showed AUC values of approximately 0.85–0.88 for detecting Ft < 20 ng/mL. Similar findings were observed for Ft < 20 with TSAT < 20. Cutoff values and their 95% confidence intervals were estimated using bootstrap resampling, reflecting the variability in threshold selection. At the Youden‐optimal cutoff, serum hepcidin and urinary hepcidin/creatinine showed comparable diagnostic performance across IDA, Ft < 20 ng/mL, and Ft < 20 ng/mL with TSAT < 20%, with sensitivities of 0.746–0.912 and specificities of 0.737–0.920. Both PPVs and NPVs—which are influenced by disease prevalence—ranged from 0.356 to 0.785 and from 0.830 to 0.970, respectively. The corresponding likelihood ratios were 3.465–9.594 for LR+ and 0.120–0.306 for LR−. Both markers demonstrated high NPVs (0.830–0.972), supporting their potential as rule‐out or triage biomarkers (Table 5). Sensitivity analyses using inverse probability weighting were conducted to assess the impact of missing urinary hepcidin data, and these analyses yielded AUC values that were very similar to the original analyses, with only small differences across outcomes (Supporting Information S1: Table S4).

**TABLE 5:** Internal validation of diagnostic performance using athlete‐level cluster bootstrap resampling.

Analysis	N samples	N athletes	N events^a^	Cutoff^b^ 95% CI	AUC 95% CI	Sensitivity 95% CI	Specificity 95% CI	PPV 95% CI	NPV 95% CI	LR+ 95% CI	LR− 95% CI
Serum hepcidin for IDA	334	220	38	2.781 (0.946–4.667)	0.905 (0.832–0.967)	0.816 (0.429–1.000)	0.811 (0.581–0.955)	0.356 (0.170–0.667)	0.972 (0.920–1.000)	4.312 (2.209–13.005)	0.227 (0.000–0.636)
Urinary hepcidin/creatinine for IDA	155	149	17	4.497 (2.057–4.497)	0.853 (0.668–1.000)	0.765 (0.181–1.000)	0.920 (0.860–1.000)	0.542 (0.200–1.000)	0.969 (0.881–1.000)	9.594 (3.467–33.006)	0.256 (0.000–0.833)
Serum hepcidin for ferritin < 20 ng/mL	334	220	134	4.184 (4.068–6.837)	0.850 (0.791–0.904)	0.746 (0.630–0.875)	0.830 (0.587–0.897)	0.746 (0.565–0.837)	0.830 (0.746–0.900)	4.390 (2.090–6.912)	0.306 (0.178–0.449)
Urinary hepcidin/creatinine for ferritin < 20 ng/mL	155	149	51	28.876 (14.559–37.867)	0.880 (0.806–0.953)	0.838 (0.533–0.936)	0.790 (0.609–1.000)	0.785 (0.625–1.000)	0.842 (0.650–0.935)	3.992 (2.175–19.996)	0.205 (0.077–0.500)
Serum hepcidin for ferritin < 20 ng/mL and TSAT < 20%	334	220	113	4.095 (1.684–4.095)	0.895 (0.830–0.947)	0.912 (0.500–0.967)	0.737 (0.657–0.957)	0.470 (0.361–0.793)	0.970 (0.872–0.988)	3.465 (2.490–14.281)	0.120 (0.047–0.535)
Urinary hepcidin/creatinine for ferritin < 20 ng/mL and TSAT < 20%	155	149	37	16.737 (6.485–31.545)	0.857 (0.768–0.941)	0.811 (0.428–1.000)	0.788 (0.521–0.930)	0.545 (0.300–0.722)	0.930 (0.812–1.000)	3.827 (1.810–6.922)	0.240 (0.000–0.652)

^a^ *N* events indicates the number of cases meeting the endpoint definition.

^b^ Serum hepcidin is expressed in ng/mL, and urinary hepcidin/creatinine is expressed in μg/g·Cr.

CRP values were low in this cohort, with only one sample exceeding 0.3 mg/dL, and only three samples had an eGFR < 60 mL/min/1.73 m^2^. Excluding these samples did not materially change the results of the ROC analyses (Supporting Information S1: Table S5). The AUC for serum hepcidin remained 0.905, and that for urinary hepcidin/creatinine remained 0.855, suggesting that, within this cohort, diagnostic performance was not substantially influenced by mild variations in inflammation or renal function.

### 3.4 Transcutaneous Hb as an Indicator of IDA

**FIGURE 6:**
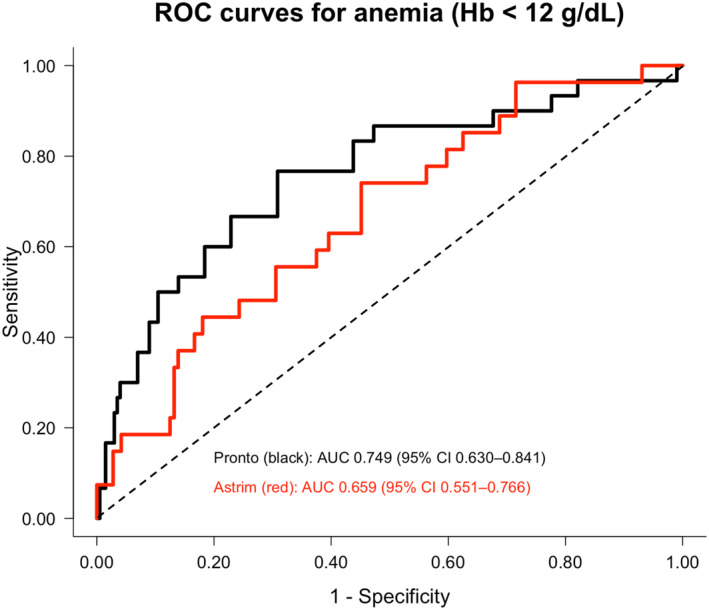
Receiver operating characteristic (ROC) curves of transcutaneous Hb measurements for detecting anemia. ROC curves for pronto (black) and astrim (red) for detecting anemia (Hb < 12 g/dL) are shown. ROC curves represent apparent performance. AUC values and 95% confidence intervals were estimated using athlete‐level cluster bootstrap resampling.

Transcutaneous Hb values measured by Pronto were significantly correlated with invasive Hb levels (*β* = 0.334, 95% CI 0.230–0.437, *p* < 0.001). Astrim measurements also showed a weaker but significant correlation with invasive Hb (*β* = 0.149, 95% CI 0.049–0.249, *p* = 0.004) (Supporting Information S1: Table S6 and Figure S2). The AUC of Pronto for anemia was approximately 0.75, while that of Astrim was approximately 0.66 (Table 6) (Figure 6). The diagnostic performance of transcutaneous Hb measurements (Pronto and Astrim) for anemia was moderate, whereas urinary hepcidin/creatinine showed higher AUC values and more favorable likelihood ratios for detecting iron deficiency–related conditions, including IDA, Ft < 20 ng/mL, and Ft < 20 ng/mL with TSAT < 20%.

**TABLE 6:** Internal validation of diagnostic performance of transcutaneous Hb using athlete‐level cluster bootstrap resampling.

Analysis	*N* samples	*N* athletes	Cutoff 95% CI	AUC 95% CI	Sensitivity 95% CI	Specificity 95% CI	PPV 95% CI	NPV 95% CI	LR+ 95% CI	LR− 95% CI
Pronto for Hb < 12 g/dL	231	149	13.300 (12.800–14.000)	0.749 (0.598–0.889)	0.667 (0.250–0.917)	0.756 (0.444–0.903)	0.290 (0.118–0.429)	0.938 (0.864–0.982)	2.735 (1.255–4.377)	0.441 (0.147–0.909)
Astrim for Hb < 12 g/dL	171	128	12.700 (11.000–13.500)	0.659 (0.507–0.808)	0.741 (0.111–1.000)	0.521 (0.203–0.891)	0.225 (0.071–0.385)	0.915 (0.791–1.000)	1.546 (0.630–3.030)	0.498 (0.000–1.162)

### 3.5 Oral Iron Treatment

**FIGURE 7:**
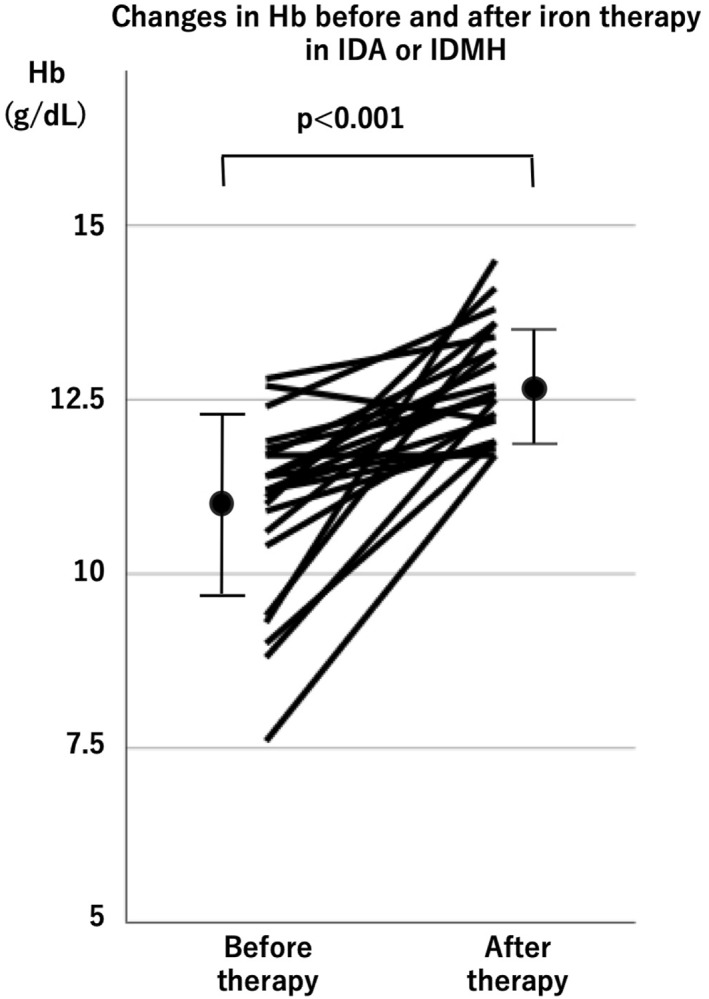
Changes in Hb before and after oral iron therapy in IDA or IDMH. Error bars indicate standard deviation. The mean Hb concentrations before and after treatment were compared using the paired student's *t* test.

Twenty‐three athletes who agreed to receive oral iron therapy at IDMH or IDA showed a significant improvement in Hb levels before and after treatment (before: 10.9 ± 1.3 vs. after: 12.7 ± 0.8 g/dL; *p* < 0.001) (Figure 7). However, two of these athletes did not experience improvement in Hb levels despite taking 100 mg of oral iron daily for 3 months.

## 4 Discussion

Urinary hepcidin, which can be measured non‐invasively, demonstrated strong potential as an indicator of iron status in female athletes. In this study, urinary hepcidin/creatinine correlated closely with serum hepcidin and showed a positive association with Ft levels. Consistent with these relationships, pairwise comparisons revealed progressive declines in both serum hepcidin and urinary hepcidin/creatinine across the spectrum from iron replete status to iron deficiency anemia. Furthermore, urinary hepcidin exhibited good diagnostic performance in ROC analyses for iron deficiency and iron deficiency anemia. Notably, its high NPV suggest that urinary hepcidin may serve as a practical rule‐out or triage biomarker, offering a useful non‐invasive tool for screening iron deficiency in athletic populations.

### 4.1 Urinary Hepcidin as a Non‐Invasive Test

Considering the burden placed on athletes and the limited availability of trained personnel to perform venipuncture in schools and sports clubs, a non‐invasive urine‐based test capable of detecting anemia and iron deficiency would be highly valuable. One such non‐invasive method is transcutaneous Hb measurement, and in this study we evaluated its utility using two different models. However, the AUC values for transcutaneous Hb in ROC analyses for anemia defined by invasive Hb measurement (Hb < 12 g/dL) were not as favorable as those observed for urinary hepcidin in detecting iron deficiency and iron deficiency anemia. Although several studies have reported the usefulness of transcutaneous Hb measurement and demonstrated good correlations with invasive Hb values (Bhat et al. 2016; Paksu et al. 2016), strong correlations are typically observed in datasets that include cases with markedly reduced Hb levels (García‐Soler et al. 2017). In contrast, most female athletes with IDA in our cohort had only mildly reduced Hb levels (10.5–11.9 g/dL), making mild IDA more difficult to detect using transcutaneous Hb (Margulies et al. 2021). Furthermore, although most anemia in female athletes is attributable to iron deficiency, it was not surprising that no correlation was observed between Ft—which correlated with urinary hepcidin—and transcutaneous Hb values (Supporting Information S1: Figure S3), further suggesting that transcutaneous Hb measurement is not suitable for detecting iron deficiency in this population.

Hepcidin is a small peptide that readily passes through the glomerulus. In this study, urinary hepcidin was present at high concentrations, and in many samples its concentration exceeded that of serum hepcidin (data not shown). Urinary hepcidin showed a strong correlation with serum hepcidin, indicating that it reflects systemic iron dynamics in athletes. Urinary hepcidin may reflect systemic iron dynamics in populations with largely preserved renal function and minimal systemic inflammation, as observed in the present cohort. However, given the limited number of participants with impaired renal function or elevated inflammatory markers, the robustness of urinary hepcidin under such conditions requires further investigation. Although early‐morning urine and samples collected during health examinations differed slightly in the time required for freezing and storage, urinary hepcidin/creatinine demonstrated good diagnostic performance for iron deficiency and iron deficiency anemia when samples were frozen within 6 hours of collection. Its high negative predictive values further suggest that urinary hepcidin may serve as a practical rule‐out biomarker. Anderson et al. also reported that storing urine samples overnight at 4°C before freezing had no appreciable effect on hepcidin measurements (Anderson et al. 2010). Taken together, these findings indicate that urinary hepcidin testing for the diagnosis of iron deficiency and iron deficiency anemia can be feasibly implemented in various settings, including schools and sports clubs.

### 4.2 Significance of Hepcidin Testing for Iron Deficiency and Iron Deficiency Anemia

In the athletes studied in this study, blood hepcidin and urinary hepcidin showed a good positive correlation with Ft levels. Furthermore, when blood hepcidin and urinary hepcidin levels were significantly reduced, Hb levels were also reduced (Supporting Information S1: Figure S4). In the early stages of iron deficiency, serum Ft, which reflects stored iron, begins to decline, and as iron deficiency progresses, reductions in MCV and MCH emerge, eventually leading to a decrease in hemoglobin as erythropoiesis becomes increasingly impaired (Camaschella 2019). The results of the present study on the relationship between hepcidin and Ft levels and the relationship between hepcidin and Hb levels are thought to provide good insight into this progress. There are several reports on the relationship between hepcidin and Ft. In non‐anemic athletes, a statistically significant difference was observed in urinary hepcidin between athletes with Ft of 15–30 ng/mL and those with Ft of 30–50 ng/mL (Borrione et al. 2011). In addition, in a study of iron deficiency in children, a positive correlation was observed between Ft and hepcidin, and serum hepcidin was reported to be effective in detecting iron deficiency in children (Choi et al. 2012). It has also been reported that urinary hepcidin is positively correlated with Ft in detecting early iron deficiency in children, and urinary hepcidin may be a useful noninvasive test (Sanad and Gharib 2011).

### 4.3 Significance of Hepcidin Testing Other Than Absolute Iron Deficiency

In this study, 2%–3% of athletes were found to be anemic, yet their anemia was not attributable to iron deficiency; instead, they were classified as having non–iron‐deficiency anemia (NIDA), with hemoglobin levels below 12 g/dL despite Ft levels above 30 ng/mL. Most of these athletes did not have low hepcidin levels (Supporting Information S1: Figure S4). Furthermore, among the athletes classified as having iron deficiency anemia with Ft levels below 30 ng/mL and Hb levels below 12 g/dL, a small number did not respond to oral iron supplements, suggesting that causes other than absolute iron deficiency may be involved. Although iron deficiency is the main cause of anemia in athletes, other causes have also been reported. Inflammatory anemia is one of these (McClung et al. 2013; Skarpańska‐Stejnborn et al. 2015), and overreaching and RED are also thought to be related to anemia (Tabata et al. 2024; Ziemann et al. 2013). Increased hepcidin levels due to inflammation inhibit the absorption of iron from the digestive tract and the release of iron from stored iron, and impaired iron utilization is thought to be the cause of inflammatory anemia (Nemeth, Rivera, et al. 2004; Nemeth, Tuttle, et al. 2004). Inflammatory anemia is generally characterized by an increase in hepcidin and no decrease in Ft levels, but it has also been noted that inflammation can increase hepcidin and decrease Ft levels (Ziemann et al. 2013). In most athletes, a positive correlation is observed between Ft and hepcidin levels, but there are also cases where this correlation does not hold. Therefore, it will be necessary to further examine what hepcidin levels truly represent in the context of iron metabolism in athletes, rather than viewing them simply as a surrogate marker for Ft levels, especially given that other biomarkers—such as soluble transferrin receptor and reticulocyte hemoglobin—are considered useful for distinguishing absolute iron deficiency from inflammation‐related functional iron deficiency (Mast et al. 2002; Punnonen et al. 1997).

### 4.4 Possibility of Screening for IDA and Iron Deficiency in Athletes Using the Urinary Hepcidin Test

It is known that iron deficiency and IDA are common among female athletes (Clénin et al. 2015; Sinclair and Hinton 2005). Sinclair et al. reported that 10% of female athletes were IDA when they defined anemia as Hb less than 12 g/dL and Ft less than 16 ng/mL (Sinclair and Hinton 2005). In our data, 15% of female athletes were anemic, but because we defined IDA as Hb < 12 g/dL and Ft < 30 ng/mL, the percentage may be slightly higher than they reported. If the analysis had been conducted using the same criteria, the proportions might have been roughly the same. It is also said that many female athletes are iron deficient even if they are not anemic (Sinclair and Hinton 2005). There are various theories about the Ft value that defines iron deficiency (Borrione et al. 2011; Clénin et al. 2015; Keller et al. 2024; Rubeor et al. 2018). Importantly, previous work has demonstrated that the prevalence of iron deficiency varies markedly depending on the Ft cutoff applied. For example, Coates et al. showed that the proportion of elite endurance athletes classified as iron deficient differed substantially when thresholds such as 20, 30, or 35 ng/mL were used, highlighting the difficulty of interpreting Ft in athletic populations (Coates et al. 2017). In our dataset, when iron deficiency was defined as Ft < 30 ng/mL, more than half of the participants met this criterion. Even if athletes are not anemic, iron deficiency in their Ft levels below 20 ng/mL can affect their performance (Keller et al. 2024; Rubeor et al. 2018). Therefore, athletes with Ft levels below 20 ng/mL may warrant further nutritional assessment and follow‐up, depending on the clinical context and confirmatory evaluation. In such cases, consultation with a registered dietitian could be considered to support dietary optimization and, where appropriate, iron supplementation.

At schools and sports clubs, where trained personnel for blood collection are not always available, conducting frequent hematological assessments for large numbers of athletes can be challenging. Given the high negative predictive values observed for the urinary hepcidin/creatinine ratio, this measure may serve as an effective rule‐out tool for iron deficiency and iron deficiency anemia, allowing blood tests to be reserved for situations in which they are truly necessary, given that venipuncture places a practical burden on athletes and requires substantial logistical preparation in real‐world sport settings. Regular hematological screening two to three times per year is generally recommended for female athletes, with even more frequent monitoring advised for those involved in endurance sports or with a history of anemia. Incorporating urinary testing into routine monitoring may reduce the burden of repeated blood sampling and enable earlier detection of iron deficiency during vulnerable periods, such as after discontinuation of iron supplementation or during pre‐season and in‐season phases. Collectively, these considerations underscore the practical value of urinary hepcidin/creatinine measurement as a non‐invasive approach for managing iron status in female athletes. However, because urinary hepcidin measurements were available only in a subset of participants, the proposed urinary cutoff values should be interpreted cautiously. Further external validation in larger and independent cohorts is warranted before broader clinical implementation. In addition, future studies should adopt more standardized urine sampling protocols, such as first‐morning void collection and/or fixed post‐exercise sampling intervals, to better quantify biological variability related to exercise timing, menstrual cycle phase, and hydration status.

## 5 Limitations

This study has several limitations. First, urinary hepcidin measurements were available only for a subset of samples, and the availability of these data differed by sport discipline and age, suggesting a potential risk of selection bias. Second, detailed information on factors known to influence hepcidin levels—such as timing of exercise, hydration status, and menstrual cycle phase—was not collected, and future studies with standardized protocols are needed to address these variables. Third, the effects of oral iron therapy using a uniform dosage and duration were not examined; therefore, the proportion of athletes who improved and the magnitude of improvement remain unclear. Fourth, other relevant biomarkers of iron status, including soluble transferrin receptor and reticulocyte Hb, were not assessed. Fifth, the optimal implementation strategy for incorporating urinary hepcidin into athlete screening programs has yet to be determined. Finally, the study population consisted exclusively of female athletes from a specific environment, and external validation in other athletic populations is required to confirm the generalizability of our findings. In addition, the number of participants with impaired renal function or elevated inflammatory markers was very limited, which restricts our ability to draw definitive conclusions regarding the influence of these factors on urinary hepcidin.

## 6 Conclusions and Practical Applications

Hepcidin, the key hormone regulating iron metabolism, plays an important role in athletes and is considered a valuable biomarker. It is detectable not only in blood but also in urine at measurable concentrations, and urinary levels correlate with serum hepcidin as well as with serum Ft. In this study, both serum and urinary hepcidin showed a stepwise decline across four iron‐status groups—from iron replete to iron deficiency anemia—demonstrating their ability to reflect progressive iron depletion. Urinary hepcidin also exhibited good diagnostic performance in ROC analyses for iron deficiency and iron deficiency anemia, and its high negative predictive values indicate potential utility as a practical rule‐out or triage biomarker.

Because frequent blood sampling is often difficult in school and club settings where trained personnel are not always available, urinary hepcidin offers a non‐invasive alternative that may facilitate screening for iron deficiency and iron deficiency anemia in athletes. Furthermore, given that inflammation increases hepcidin while lowering Ft, urinary hepcidin may serve not merely as a surrogate for Ft but also as a biomarker with broader applications in monitoring iron metabolism under various physiological conditions. These findings highlight the potential value of urinary hepcidin measurement as a practical and accessible tool for athlete health management. However, the proposed diagnostic cutoffs were derived from internally validated analyses and should be considered preliminary; external validation in independent populations is required before widespread implementation.

## Funding

This work was supported by the Ministry of Education, Culture, Sports, Science and Technology of Japan (23K10622) and the Japan Sports Agency Female Athlete Support Project (2022 and 2023).

## Ethics Statement

The study was conducted according to the guidelines of the Declaration of Helsinki and approved by the Ethics Committee of Niigata University of Health and Welfare (18637–210618; approved on 18 June 2021).

## Consent

Informed consent was obtained from all subjects involved in the study. No individual and personal data were shown in this manuscript.

## Conflicts of Interest

The authors declare no conflicts of interest.

## Supporting information


Supporting Information S1

## Data Availability

Research data are not shared.
