# Supplementary material for: Potential Usefulness of Urinary Hepcidin Measurement for Iron Deficiency Anemia in Female Athletes
Source: Eur J Sport Sci. 2026 May 22;26(6):e70198. doi: 10.1002/ejsc.70198 (PMC13240275; doi:10.1002/ejsc.70198)
Supplement: Supplementary file 1 — Supporting Information S1 [file EJSC-26-e70198-s001.docx]

Supplementary Figure S1.


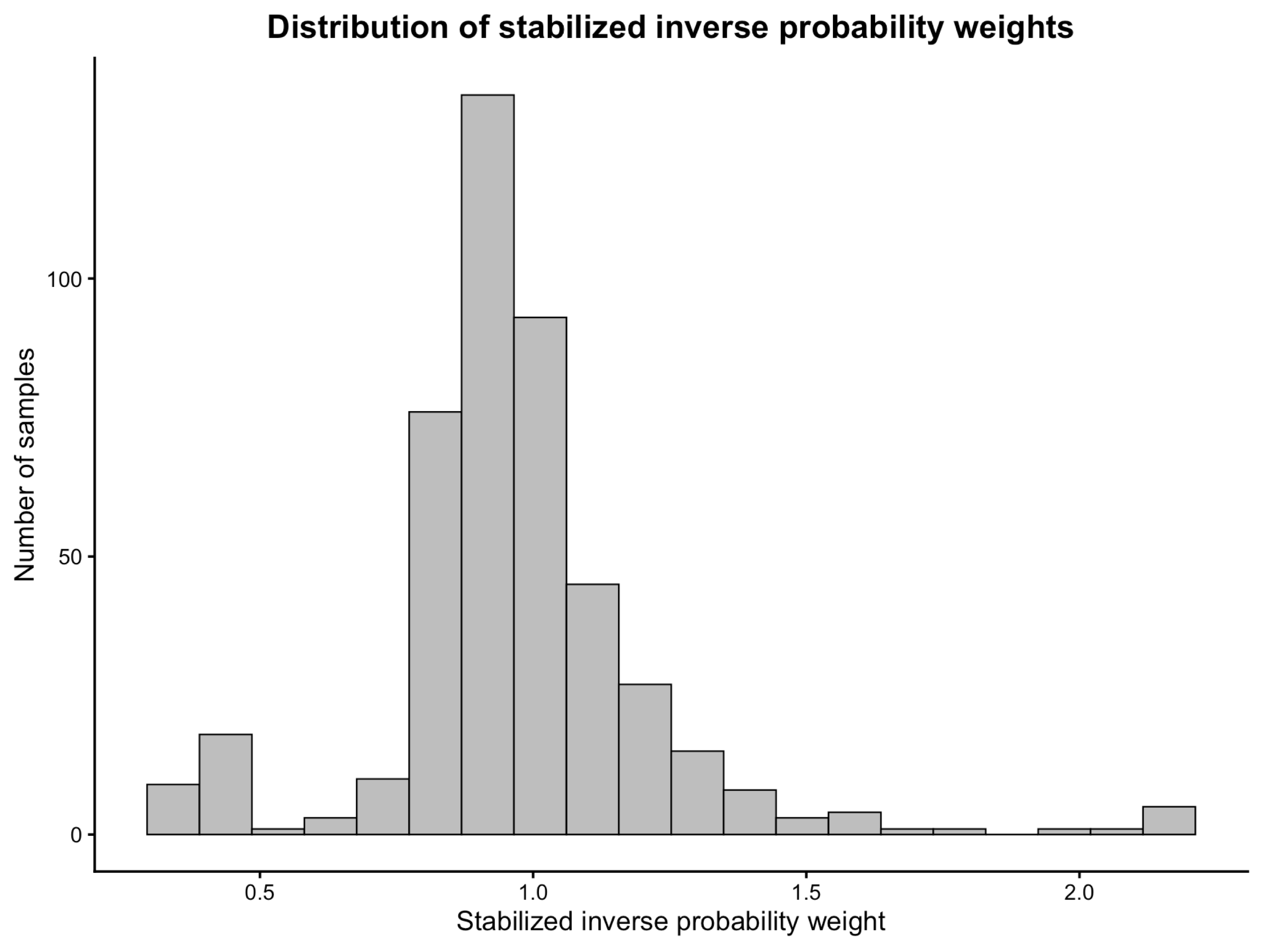


Distribution of stabilized inverse probability weights used in sensitivity.

The histogram shows the distribution of stabilized weights derived from the propensity score model for urinary hepcidin availability. The weights were well distributed without extreme values, indicating adequate model stability.

Supplementary Figure S2.


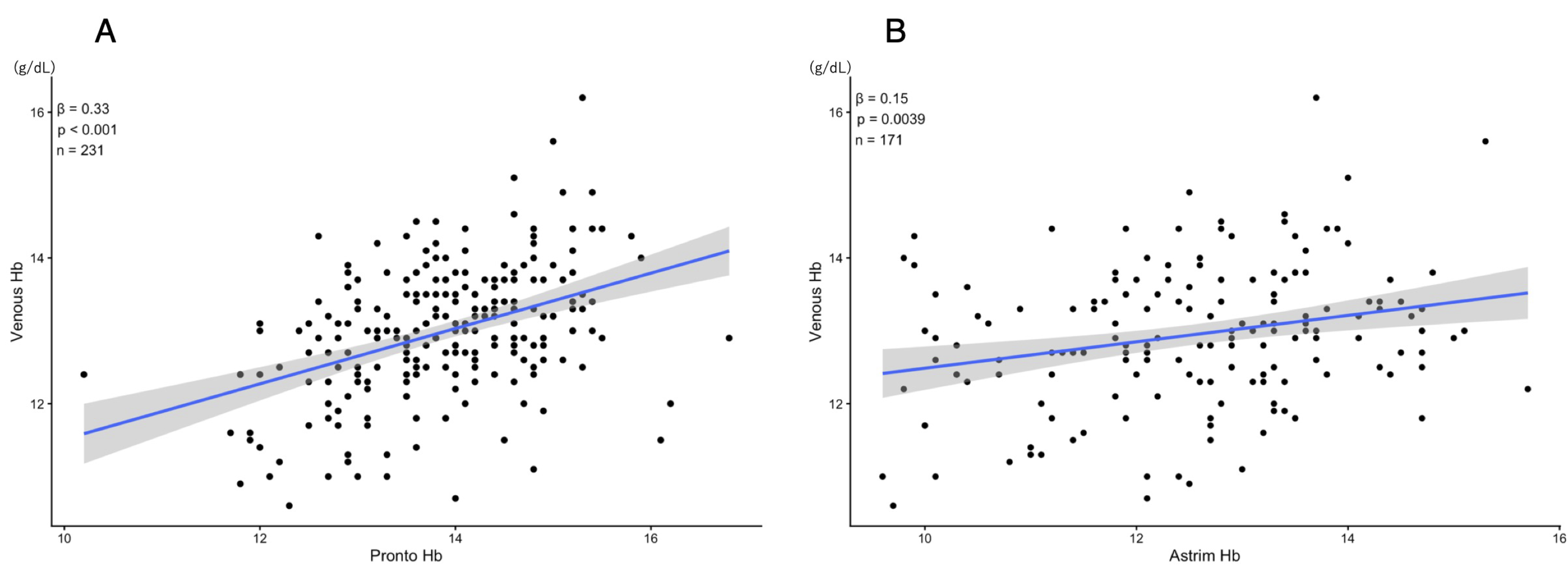


Associations between biomarkers.

(A) Relationship between Pronto Hb and Venous Hb.

(B) Relationship between Astrim Hb and Venous Hb.

Lines represent fitted values from mixed-effects linear regression models with random intercepts for each athlete. Shaded areas indicate 95% confidence intervals.

Supplementary Figure 3


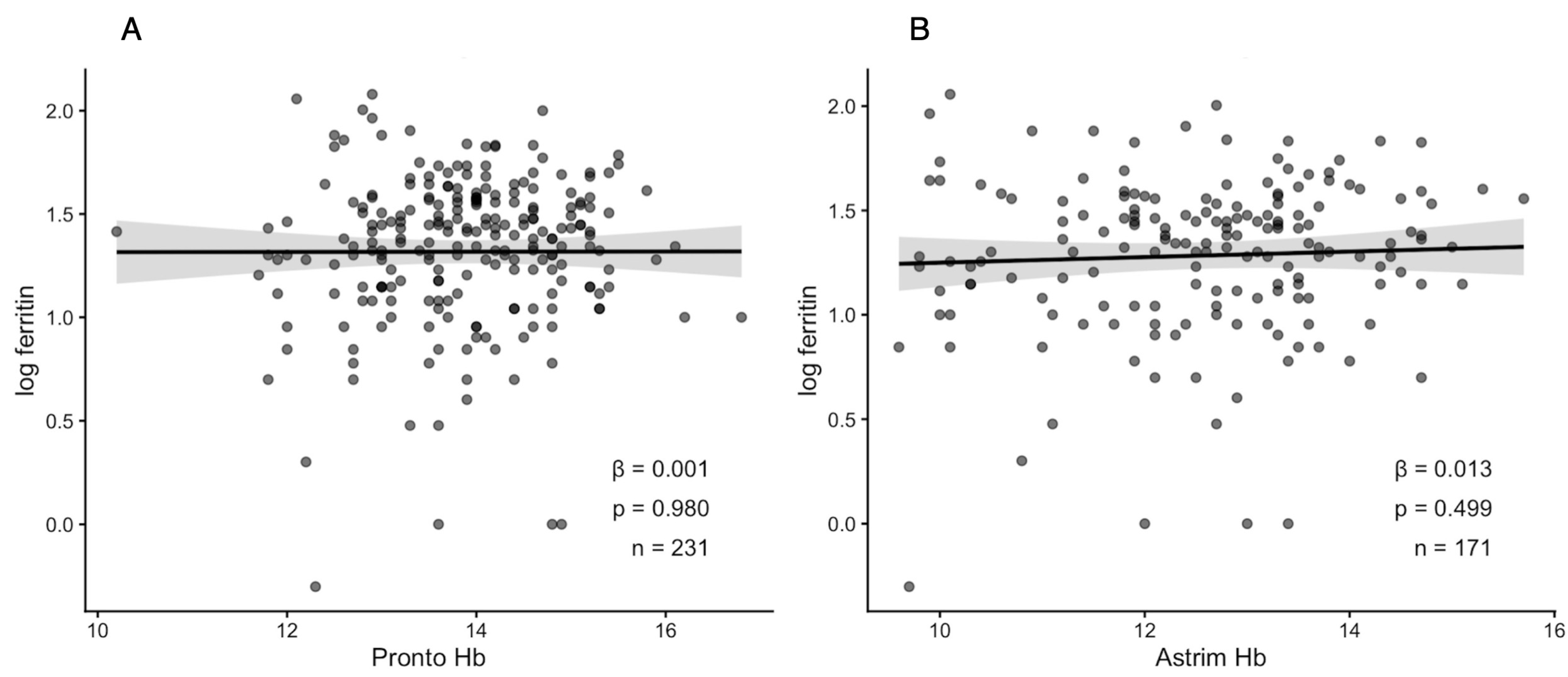


Associations of transcutaneous Hb measurements with log Ft.

(A) Relationship between Pronto Hb and log Ft.

(B) Relationship between Astrim Hb and log Ft.

Lines represent fitted values from mixed-effects linear regression models with random intercepts for each athlete, and shaded areas indicate 95% confidence intervals.

Supplementary Figure 4


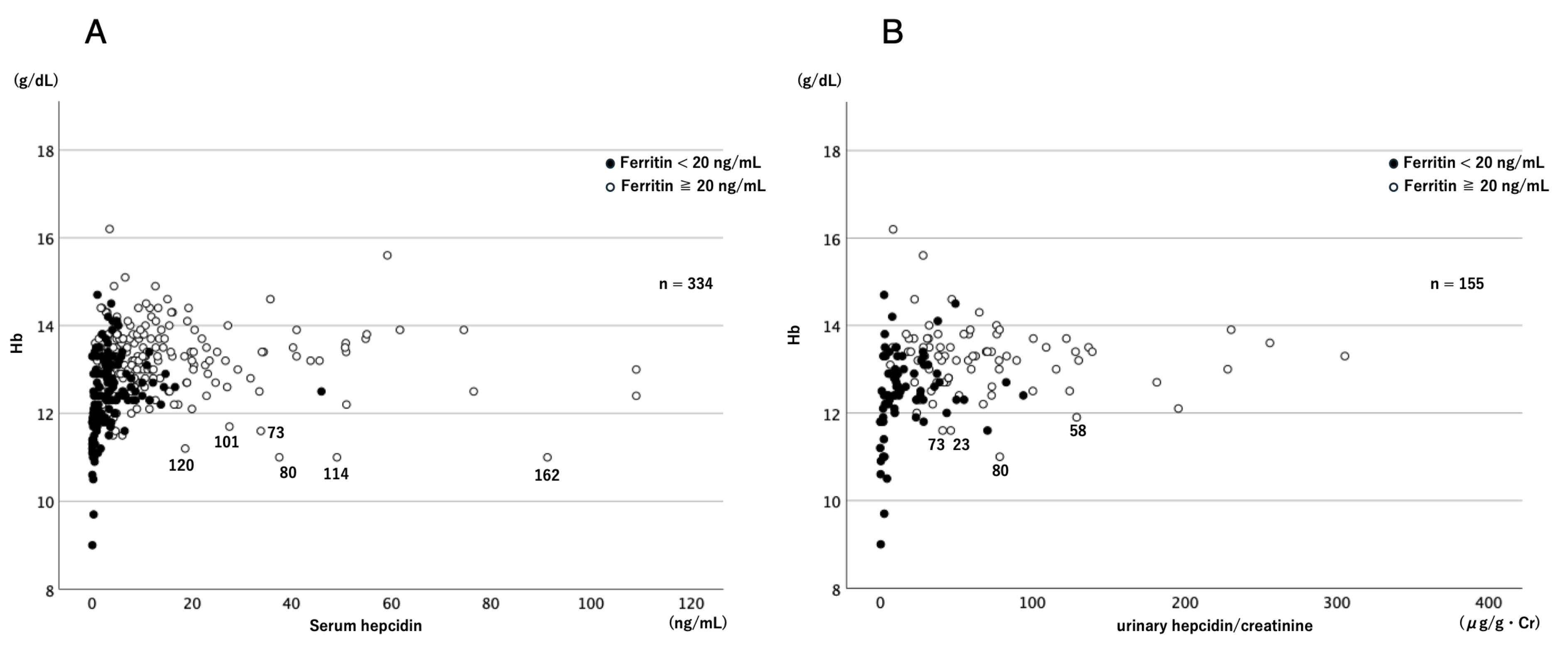


Relationship between serum hepcidin and Hb (A). Relationship between urinary hepcidin/creatinine and Hb (B). Numbers represent Ft (ng/mL). Closed circles (●) represent Ft < 20 ng/mL, whereas open circles (○) represent Ft ≥ 20 ng/mL.

Supplementary Table 1

Validation performance of the urinary hepcidin ELISA assay

Supplementary Table 2

Factors associated with availability of urinary hepcidin measurements using Firth’s penalized logistic regression

Supplementary Table 3

Conventional diagnostic performance analysis without internal validation

Supplementary Table 4

Sensitivity analysis using inverse probability weighting

Supplementary Table 5

AUC without high CRP and low eGFR

Supplementary Table 6

Associations of Pronto and Astrim Hb measurements with venous Hb evaluated using mixed-effects linear regression models
